# Does tumor location affect irreversible electroporation outcomes in pancreatic cancer? A head-to-head comparative cohort study

**DOI:** 10.3389/fonc.2026.1890752

**Published:** 2026-08-19

**Authors:** Tiankuan Li, Xiaoyu Liu, Qungang Shan, Jingfeng Li, Wei Huang, Xiaoheng Hu

**Affiliations:** 1 Department of Interventional Radiology, Wuxi branch of Ruijin Hospital Shanghai, Wuxi, Jiangsu, China; 2 Department of Radiology, Ruijin Hospital, Shanghai Jiao Tong University School of Medicine, Shanghai, China

**Keywords:** advanced pancreatic cancer, event-free survival, irreversible electroporation, overall survival, tumor location

## Abstract

**Objective:**

Irreversible electroporation (IRE) is a promising non-thermal ablative modality for advanced pancreatic cancer (APC). However, whether tumor location influences outcomes remain unclear due to distinct anatomical relationships between the pancreatic head/neck and body/tail. This study compared the safety, efficacy, and survival outcomes of IRE between patients with pancreatic head/neck and body/tail tumors.

**Methods:**

We analyzed a real-world cohort of 63 consecutive patients with advanced pancreatic cancer (comprising primary, post-resection recurrent, and oligometastatic diseases) who underwent percutaneous CT-guided IRE between March 2018 and July 2024. Patients were stratified into the head/neck group (n=39) and the body/tail group (n=24). Baseline characteristics, procedural parameters, adverse events, overall survival (OS), event-free survival (EFS), and tumor marker changes (CA19-9/CEA) were compared.

**Results:**

Median age was 66 years (head/neck) versus 61 years (body/tail) (*P* = 0.468). The body/tail group had significantly higher body weight (median 56 vs. 47 kg, *P* = 0.021) and BMI (median 20.19 vs. 17.99 kg/m², *P* = 0.021). No significant differences were observed in IRE procedural parameters. Elevated serum amylase was the most common adverse event (61.5% vs. 58.3%, *P* = 1.000), and complication rates were comparable. Median OS from surgery was 8.7 versus 9.37 months (*P* = 0.788), while OS from diagnosis was 22.80 versus 21.32 months (*P* = 0.948). Median EFS was 6.00 versus 6.25 months (*P* = 0.357). No significant differences were found in 1-month post-IRE CA19–9 or CEA ratio changes (*P* = 0.783 and *P* = 0.295, respectively).

**Conclusions:**

In this cohort of patients with relatively low BMI, percutaneous CT-guided IRE demonstrated comparable safety and efficacy between pancreatic head/neck and body/tail locations. Tumor location alone may not limit IRE feasibility; however, caution is needed when generalizing these findings to Western populations with higher BMI, and larger prospective studies are warranted.

## 1 Introduction

Pancreatic cancer remains one of the most lethal malignancies worldwide, with a 5-year overall survival rate of approximately 12% across all stages (1, 2). More than 80% of patients are present with advanced or metastatic disease at the time of diagnosis, rendering curative surgical resection infeasible (3). For patients with advanced pancreatic cancer (APC), which accounts for approximately 30-40% of newly diagnosed cases, the standard-of-care treatment includes systemic chemotherapy with or without radiation therapy (4, 5). However, the median overall survival for APC patients treated with chemotherapy alone remains limited to 11–14 months with traditional monotherapy, underscoring the urgent need for effective local therapeutic strategies (6, 7).

Irreversible electroporation (IRE), also referred to as NanoKnife ablation, is a non-thermal ablative technique that employs high-voltage, low-energy direct current pulses to create permanent nanopores in the cell membrane, leading to disruption of cellular homeostasis and subsequent apoptotic cell death (8, 9). Unlike conventional thermal ablation modalities such as radiofrequency ablation (RFA) and microwave ablation (MWA), IRE selectively targets the lipid bilayer while preserving the structural integrity of the extracellular matrix, blood vessels, and bile ducts — structures that are particularly vulnerable to thermal damage in the pancreas (10, 11). This unique property makes IRE especially suitable for pancreatic tumors encasing or abutting critical peripancreatic vasculature, including the superior mesenteric artery, celiac trunk, and portal vein (12).

Over the past decade, a growing body of clinical evidence has demonstrated the safety and efficacy of IRE for APC. The Phase I/II PANFIRE study established the feasibility and safety of percutaneous CT-guided IRE in 25 patients with advanced pancreatic cancer, reporting a median OS of 11 months from the time of IRE (13). The subsequent multicenter PANFIRE-2 Phase II trial confirmed these findings in a larger cohort of 50 patients, with a median OS of 17 months from diagnosis and an acceptable adverse event profile (14). Additional single-center experiences and systematic reviews have corroborated these observations, consistently showing that IRE can provide meaningful survival prolongation with manageable perioperative risks (15–17).

An important but relatively underexplored question in the context of IRE for pancreatic cancer is whether tumor location within the pancreas — specifically, the head/neck versus the body/tail — affects treatment outcomes. The pancreas is anatomically and functionally heterogeneous, with the head region intimately related to the duodenum, common bile duct, and pancreatic duct confluence, while the body and tail are situated in the retroperitoneum with distinct vascular relationships (18). These anatomical differences may theoretically influence procedural complexity, probe placement feasibility, ablation completeness, and the spectrum of perioperative complications.

To date, no dedicated studies have systematically compared IRE outcomes between pancreatic head/neck and body/tail tumors in a head-to-head fashion. Most published series have reported aggregate outcomes without stratifying results by tumor subsite, leaving an important gap in the literature that may have direct implications for patient selection, procedural planning, and informed consent discussions. Elucidating whether tumor location independently influences safety and efficacy endpoints is critical for optimizing the application of this increasingly utilized ablative modality.

In the present study, we performed a retrospective analysis of a real-world cohort comprising 63 consecutive patients with advanced pancreatic cancer—including primary, post-resection recurrent, and oligometastatic diseases— who underwent percutaneous CT-guided IRE at our center. Reflecting everyday clinical practice, this heterogeneous real-world setting allows us to evaluate whether anatomical location independently influences procedural feasibility and clinical outcomes under broad clinical conditions. Patients were stratified into two groups based on tumor location: the pancreatic head/neck group (n = 39) and the pancreatic body/tail group (n = 24). We comprehensively compared baseline patient characteristics, procedural parameters, perioperative adverse events, survival outcomes, and tumor marker dynamics between the two groups. We hypothesized that IRE would demonstrate comparable safety and efficacy profiles irrespective of pancreatic tumor location.

## 2 Materials and methods

### 2.1 Study design and patient population

This was a retrospective, single-center cohort study conducted at the Department of Radiology, Ruijin Hospital. The study protocol was approved by the Institutional Review Board of Ruijin Hospital (Approval number: 2024-421), and the requirement for written informed consent was waived due to the retrospective nature of the analysis. All procedures performed were in accordance with the ethical standards of the institutional research committee and with the Helsinki Declaration and its later amendments.

We consecutively screened and reviewed the medical records of all patients with histopathologically confirmed pancreatic cancer who underwent percutaneous CT-guided IRE between March 2018 and July 2024 to minimize potential selection bias. The inclusion criteria were: (1) age ≥ 18 years; (2) histopathologically confirmed pancreatic ductal adenocarcinoma (PDAC) or other histological variants; (3) All 63 eligible patients with mixed advanced or recurrent pancreatic cancer, deemed surgically unresectable by multidisciplinary tumor boards, were enrolled for final analysis. Locally advanced cases were inoperable due to multiorgan metastatic. Recurrent tumors arose from prior resections matched to primary sites: pancreaticoduodenectomy for pancreatic head/neck lesions and distal pancreatectomy for body/tail lesions. For patients presenting with stable, oligometastatic disease, IRE was indicated solely for local tumor control to prevent or palliate primary-tumor-related complications (such as severe pain or biliary/duodenal obstruction) and facilitate the continuation of systemic therapy. All patients underwent a rigorous multidisciplinary tumor board (MDT) review and provided written informed consent, explicitly acknowledging the palliative, local-control nature of the IRE procedure; (4) Eastern Cooperative Oncology Group (ECOG) performance status of 0-2; (5) adequate coagulation function (international normalized ratio [INR] ≤ 1.5, platelet count ≥ 50 × 10^9/L); and (6) complete medical records and post-procedural follow-up data. The exclusion criteria were: (1) uncontrollable cardiac arrhythmia or presence of a cardiac pacemaker; (2) active infection refractory to antibiotic therapy; (3) pregnancy; and (4) known allergy to anesthetic agents or contrast media.

A total of 63 patients met the eligibility criteria and were included in the final analysis. Patients were categorized into two groups based on tumor location as determined by preoperative contrast-enhanced computed tomography (CT) and/or magnetic resonance imaging (MRI): the pancreatic head/neck group (n = 39), comprising tumors located in the head, uncinate process, or neck of the pancreas, and the pancreatic body/tail group (n = 24), comprising tumors located in the body or tail of the pancreas.

### 2.2 IRE procedure

All IRE procedures were performed using the NanoKnife System (AngioDynamics, Latham, NY, USA) under general anesthesia with complete neuromuscular blockade to prevent muscle contractions induced by the electrical pulses. A cardiac synchronized delivery mode was employed, with pulse delivery triggered by the R-wave of the electrocardiogram to minimize the risk of arrhythmia (19).

Procedures were performed under CT guidance (Siemens Healthineers, Forchheim, Germany). Following induction of general anesthesia, the patient was positioned supine or in a slight lateral decubitus position depending on the tumor location. A preliminary CT scan was acquired to confirm the tumor position and plan the optimal needle trajectory. Under sterile conditions, two to five 19-gauge monopolar IRE electrodes were percutaneously inserted into and around the tumor using a parallel or slightly convergent configuration, with inter-electrode spacing maintained between 1.5 and 2.5 cm. The electrode tips were positioned to ensure complete coverage of the tumor volume with an adequate ablative margin of at least 0.5 cm beyond the visible tumor boundary (20).

Initially, test pulses (10 pulses at 1800 V/cm) were administered over a 70 μs period, followed by the delivery of 90 target therapeutic pulses ranging from 1500 to 3000 V/cm at a current of 20–50 A. In cases where the initial procedure was prematurely terminated prior to the completion of the 90-pulse protocol, a repeat intervention was performed. Depending on tumor size and geometry, a pull-back technique (sequential repositioning of electrodes to treat overlapping ablation zones) or electrode replacement (re-insertion of electrodes to cover different tumor regions) was employed as needed to achieve complete ablation coverage. The total IRE delivery time was defined as the cumulative duration of electrical pulse delivery across all electrode pairs. The total procedure duration was calculated from the initiation of the first CT planning scan to the removal of the last electrode. Successful IRE delivery, defined as completion of the planned treatment without intraprocedural termination due to technical failure (defined as intraprocedural uncontrollable arrhythmia, IRE hardware malfunction, or acute major hemorrhage preventing complete pulse delivery) or adverse events, was recorded for each case.

Post-procedural management included routine monitoring in the post-anesthesia care unit, serial serum amylase and bilirubin measurements, and a contrast-enhanced CT scan within 24–48 hours to assess the immediate ablation zone and screen for early complications. Patients were discharged on postoperative day 5-38, following confirmation of clinical stability and absence of major complications (21).

### 2.3 Data collection and outcome definitions

Demographic and baseline clinical data, including age, sex, height, weight, body mass index (BMI), medical comorbidities, tumor pathological type, regional lymph node invasion status, and presence of distant organ metastasis, were extracted from the electronic medical record system. Tumor diameter and volume were measured on preoperative cross-sectional imaging using the Picture Archiving and Communication System (PACS). Data on neoadjuvant therapies, including pre-IRE chemotherapy, radiotherapy, and immunotherapy, were also recorded.

Procedural data included the time from initial tumor detection to IRE surgery, number of IRE probes used, electrode deployment technique (single-pass ablation, pull-back technique, or electrode replacement), total IRE delivery time, total procedure duration, and length of hospital stay. Adjuvant treatment details, including post-IRE chemotherapy, immunotherapy, radiotherapy, and surgical resection, were documented.

Adverse events were assessed during the index hospitalization and within 90 days of the IRE procedure. All complications were graded according to the Cardiovascular and Interventional Radiological Society of Europe (CIRSE) classification system (22), which classifies adverse events into five grades: Grade 1 (no therapy required, no consequence), Grade 2 (nominal therapy, overnight admission for observation only), Grade 3 (required therapy, minor hospitalization < 48 hours), Grade 4 (major therapy, unplanned increase in level of care, prolonged hospitalization > 48 hours), and Grade 5 (permanent adverse sequelae). Specific adverse events recorded included fever, pain, bleeding, ascites, serum amylase elevation, serum bilirubin elevation, the time required for laboratory values to return to normal, and portal vein thrombosis.

The primary outcome measures were overall survival (OS), defined as the time from IRE surgery to death from any cause, and OS from the date of initial pancreatic cancer diagnosis to death. Secondary outcomes included: (1) event-free survival (EFS), defined as the time from IRE surgery to the first occurrence of local tumor progression, distant metastasis, or death; (2) the incidence and severity of IRE-related adverse events; and (3) changes in serum tumor markers, assessed as the ratio of the one-month post-IRE value to the pre-IRE value for carbohydrate antigen 19-9 (CA19-9) and carcinoembryonic antigen (CEA) (23, 24).

### 2.4 Statistical analysis

Continuous variables were assessed for normality using the Shapiro-Wilk test. As the majority of continuous variables — including total IRE delivery time and total procedure duration — were found to be non-normally distributed (P < 0.05), all continuous data are presented as median (minimum-maximum) and were compared between groups using the Mann-Whitney U test (25). Categorical variables are presented as frequencies with percentages and were compared using either the chi-squared test or Fisher’s exact test, as appropriate based on expected cell frequencies.

Survival outcomes were estimated using the Kaplan-Meier method (26), with comparisons between the head/neck and body/tail groups performed using the log-rank test. Patients who were alive at the time of the last follow-up were censored. For the three surviving patients in our cohort, right-censoring was applied with the censoring time. The median follow-up duration was calculated using the reverse Kaplan-Meier method. Number at risk tables were generated for each survival curve.

For tumor marker analysis, the CA19–9 and CEA change ratios at one-month post-IRE relative to pre-IRE levels were calculated only for patients with valid paired measurements. These ratios were compared between groups using the Mann-Whitney U test.

All statistical tests were two-sided, and a P value of less than 0.05 was considered statistically significant. Statistical analyses were performed using R Statistical Software (version 4.6.0) and SPSS (version 26.0).

For the Kaplan-Meier figures, the following visualization specifications were applied: curves were plotted as step functions with the “steps-post” interpolation method.

## 3 Results

### 3.1 Patient characteristics

A total of 63 patients with histopathologically confirmed pancreatic cancer met the inclusion criteria and were included in this study. Of these, 39 patients (61.9%) had tumors located in the pancreatic head or neck region, and 24 patients (38.1%) had tumors in the body or tail. The baseline demographic, clinical, and oncological characteristics of the study population are summarized in **Table 1**.

**Table 1:** Baseline characteristics of pancreatic cancer patients treated with irreversible electroporation.

Characteristic	Head & neck	Body & tail	P-value
Number of patients (n)	39	24	
Median age, years	66 (34-86)	61 (40-81)	0.468
Height, cm	163 (151-178)	168 (155-178)	0.141
Weight, kg	47 (35-76)	56 (34-90)	0.021
BMI	17.99 (13.11-27.25)	20.19 (14.15-30.78)	0.021
Gender			
Female, n	20(51.3%)	15(62.5%)	
Male, n	19(48.7%)	9(37.5%)	
Medical history			
Hypertension, n	14(35.9%)	5(20.8%)	
Diabetes mellitus, n	6(15.4%)	6(25.0%)	
Coronary heart disease, n	1(2.6%)	1(4.2%)	
Cerebral infarction, n	1(2.6%)	1(4.2%)	
Hyperthyroidism, n	0(0%)	2(8.3%)	
Disease status at IRE			
Primary disease, n	26 (66.7%)	18 (75.0%)	0.578
Recurrent disease, n	13 (33.3%)	6 (25.0%)	
Median tumor diameter, cm	2.98 (1.50-5.70)	3.24 (1.79-6.68)	0.671
Median tumor volume, cm³	7.75 (1.49-130.08)	6.11 (3.35-205.74)	0.763
Tumor pathological type			
Pancreatic ductal adenocarcinoma, n	38(97.4%)	23(95.8%)	
Adenosquamous carcinoma, n	1(2.6%)	0	
Malignant transformation of MCN, n	0	1(4.2%)	
Regional lymph node invasion			
Y, n	38(97.4%)	23(95.8%)	
N, n	1(2.6%)	1(4.2%)	
Distant organ metastasis			
Y, n	17(43.6%)	12(50.0%)	
N, n	22(56.4%)	12(50.0%)	
Pre-IRE Surgical Resection, n	13 (33.3%)	6 (25.0%)	
Pre-IRE Chemotherapy, n	31(79.5%)	21(87.5%)	
Pre-IRE Radiotherapy, n	3(7.7%)	1(4.2%)	
Pre-IRE Immunotherapy, n	3(7.69%)	2(8.33%)	

Except where indicated, data are expressed as number (percentage), median (minimum–maximum). BMI, Body Mass Index; MCN, Mucinous Cystic Neoplasm; IRE, Irreversible Electroporation.

The median age at the time of IRE was 66 years (range, 34-86) in the head/neck group and 61 years (range, 40-81) in the body/tail group, with no statistically significant difference between groups (*P =* 0.468). The sex distribution was comparable, with females comprising 51.3% of the head/neck group and 62.5% of the body/tail group (*P =* 0.383). Median height was 163 cm (range, 151-178) in the head/neck group versus 168 cm (range, 155-178) in the body/tail group (*P =* 0.141). Notably, body weight was significantly higher in the body/tail group (median 56 kg, range 34-90) compared to the head/neck group (median 47 kg, range 35-76; *P =* 0.021), and this difference was reflected in the BMI values, with medians of 20.19 kg/m² (range, 14.15-30.78) and 17.99 kg/m² (range, 13.11-27.25), respectively (*P =* 0.021).

Regarding medical comorbidities, the prevalence of hypertension was 35.9% in the head/neck group and 20.8% in the body/tail group (*P =* 0.205). Diabetes mellitus was present in 15.4% and 25.0% of patients, respectively (*P =* 0.345). Coronary heart disease (2.6% vs. 4.2%, *P =* 1.000), cerebral infarction (2.6% vs. 4.2%, *P =* 1.000), and hyperthyroidism (0% vs. 8.3%, *P =* 0.141) were uncommon in both groups.

The predominant pathological type was pancreatic ductal adenocarcinoma (PDAC), accounting for 97.4% (38/39) of head/neck tumors and 95.8% (23/24) of body/tail tumors (*P =* 1.000). One patient in the head/neck group had adenosquamous carcinoma, and one patient in the body/tail group had malignant transformation of a mucinous cystic neoplasm. Regional lymph node invasion was present in nearly all patients (97.4% in head/neck vs. 95.8% in body/tail; *P =* 1.000). Distant organ metastasis was documented in 43.6% of head/neck patients and 50.0% of body/tail patients, with no significant difference (*P =* 0.621). The median tumor diameter was 2.98 cm (range, 1.50-5.70) in the head/neck group and 3.24 cm (range, 1.79-6.68) in the body/tail group (*P =* 0.671). Similarly, median tumor volume did not differ significantly between groups (7.75 cm³ [range, 1.49-130.08] vs. 6.11 cm³ [range, 3.35-205.74]; *P =* 0.763).

The proportions of patients receiving pre-IRE chemotherapy (79.5% vs. 87.5%, *P =* 0.513), pre-IRE radiotherapy (7.7% vs. 4.2%, *P =* 1.000), and pre-IRE immunotherapy (7.69% vs. 8.33% *P =* 1.000) were comparable between the two groups, confirming that the treatment background prior to IRE was well balanced across tumor locations.

### 3.2 Surgical and procedural characteristics

Detailed perioperative and procedural parameters are presented in **Table 2**. The median time from initial tumor detection to IRE surgery was 12.7 months (range, 0.2-304.2) in the head/neck group and 11.8 months (range, 1.3-53.0) in the body/tail group (*P =* 0.860). The median length of hospital stay was identical between groups at 8 days (range, 5–38 for head/neck; range, 5–18 for body/tail; *P =* 0.926), which included a routine 2-day preoperative evaluation (imaging and multidisciplinary logistics), with the extended stays driven by the management of post-procedural adverse events.

**Table 2:** Details of IRE treatment.

Characteristic	Head & neck	Body & tail	P-value
Time from Tumor Detection to Surgery, months	12.7 (0.2-304.2)	11.8 (1.3-53.0)	0.860
Hospital Stay, days	8 (5-38)	8 (5-18)	0.926
Total IRE Delivery Time, minutes	9 (4-32)	8.5 (4-32)	0.898
Total Procedure Duration, minutes	70 (45-125)	73 (46-119)	0.696
Number of IRE probes used			
2, n	21 (53.8%)	14 (58.3%)	
3, n	10 (25.6%)	7 (29.2%)	
4, n	6 (15.4%)	2 (8.3%)	
5, n	2 (5.1%)	1 (4.2%)	
Single or multiple electrode placement			
Single-pass Ablation, n	24 (61.5%)	14 (58.3%)	
Pull-back Technique, n	9 (23.1%)	7 (29.2%)	
Electrode Replacement, n	6 (15.4%)	3 (12.5%)	
Post-IRE chemotherapy			
Yes, n	29 (74.36%)	19 (79.17%)	
No, n	10 (25.64%)	5 (20.83%)	
Post-IRE immunotherapy			
Yes, n	4 (10.26%)	2 (8.33%)	
No, n	35 (89.74%)	22 (91.67%)	
Post-IRE radiotherapy			
Yes, n	6 (15.4%)	2 (8.3%)	
No, n	33 (84.6%)	22 (91.7%)	
Post-IRE surgical resection			
Yes, n	1 (2.6%)	1 (4.2%)	
No, n	38 (97.4%)	23 (95.8%)	

Except where indicated, data are expressed as number (percentage), median (minimum–maximum). IRE, Irreversible Electroporation.

IRE delivery was technically successful in all 63 patients (100% procedural success rate). On the contrast-enhanced CT acquired within 24–48 hours, 100% (63/63) of patients demonstrated an adequate, well-demarcated non-enhancing immediate ablation zone that fully covered the primary tumor with acceptable ablative margins. Aside from expected mild peri-procedural edema and minimal reactive fluid collection around the ablation zone, no early devastating imaging complications (such as target vessel rupture, acute portal vein occlusion, or gastrointestinal perforation) were observed (0%). The median total IRE delivery time was 9 minutes (range, 4-32) in the head/neck group and 8.5 minutes (range, 4-32) in the body/tail group (*P =* 0.898). The median total procedure duration was 70 minutes (range, 45-125) and 73 minutes (range, 46-119), respectively (*P =* 0.696). The distribution of IRE probe number did not differ significantly between groups (*P =* 0.865). Two probes were most commonly used in both groups (53.8% of head/neck vs. 58.3% of body/tail), followed by three probes (25.6% vs. 29.2%), four probes (15.4% vs. 8.3%), and five probes (5.1% vs. 4.2%).

Regarding electrode deployment strategies, single-pass ablation without repositioning was the predominant technique in both groups (61.5% in head/neck vs. 58.3% in body/tail). The pull-back technique was employed in 23.1% and 29.2% of cases, respectively, and electrode replacement was required in 15.4% and 12.5% (*P =* 0.849 for overall comparison). These findings suggest that procedural complexity, as reflected by probe utilization patterns and electrode deployment strategies, was not disproportionately influenced by tumor location.

Post-IRE adjuvant therapies were administered to the majority of patients. Chemotherapy was received by 74.36% of head/neck patients and 79.17% of body/tail patients (*P =* 0.896). Post-IRE immunotherapy was administered less frequently in the body/tail group (8.33%) compared to the head/neck group (10.26%), although this difference did not reach statistical significance (*P =* 1.000). Post-IRE radiotherapy rates were low in both groups (15.4% vs. 8.3%, *P =* 0.692), and only one patient in each group (2.6% and 4.2%, respectively) underwent post-IRE surgical resection (*P =* 1.000).

### 3.3 Adverse events

The spectrum and severity of perioperative adverse events, classified according to the CIRSE grading system, are detailed in **Table 3**. No procedure-related mortalities occurred within 90 days of IRE in either group.

**Table 3:** Adverse events.

Characteristic	Head & neck	Body & tail	P-value	Treatment
Number of patients (n)	39	24		
Fever, n	16 (41.0%)	9 (37.5%)	1.000	Antibiotics and drainage
Grade I/II	13	8		
Grade III	2	1		
Grade IV	1	0		
Pain, n	11 (28.2%)	5 (20.8%)	0.723	Oral or intravenous analgesics
Grade I/II	11	5		
Bleeding, n	2 (5.1%)	1 (4.2%)	1.000	Drug hemostasis and/or blood transfusion
Grade I/II	2	1		
Ascites, n	3 (7.7%)	1 (4.2%)	1.000	Albumin supplementation and/or drainage
Grade I/II	3	1		
Elevated Amylase, n	24 (61.5%)	14 (58.3%)	1.000	Pancreatic enzyme inhibitors
Grade I/II	24	14		
Time for Amylase to Return to Normal, days	6 (3,30)	6 (3,9)	0.108	
Elevated Bilirubin, n	5 (12.8%)	1 (4.2%)	0.394	Antijaundice drugs
Grade I/II	5	1		
Time for Bilirubin to Return to Normal, days	38 (25,63)	35	1.000	
Portal Vein Thrombosis, n	0 (0.0%)	1 (4.2%)	0.381	Anticoagulant drugs
Grade IV	0	1		

Except where indicated, data are expressed as number (percentage), median (minimum–maximum). All classifications adopt the CIRSE complication grading system. CIRSE, Cardiovascular and Interventional Radiological Society of Europe.

The most frequently observed adverse event was transient elevation of serum amylase, which occurred in 61.5% (24/39) of head/neck patients and 58.3% (14/24) of body/tail patients (*P =* 1.000). All episodes of amylase elevation were managed proactively with pancreatic enzyme inhibitors (specifically intravenous octreotide) to suppress pancreatic exocrine secretion and prevent acute pancreatitis, resolving safely without clinical sequelae. The median time for serum amylase to return to the normal range was 6 days (range, 3-30) in the head/neck group and 6 days (range, 3-9) in the body/tail group (*P =* 0.108). All cases of amylase elevation were CIRSE Grade 1-2.

Fever was reported in 41.0% (16/39) of head/neck patients and 37.5% (9/24) of body/tail patients (*P =* 1.000). Most febrile episodes were CIRSE Grade 1-2 (13/16 in head/neck; 8/9 in body/tail), with two Grade 3 and one Grade 4 event in the head/neck group managed successfully with antibiotics and drainage. Post-procedural pain occurred in 28.2% (11/39) of head/neck patients and 20.8% (5/24) of body/tail patients (*P =* 0.723), all of which were CIRSE Grade 1–2 and controlled with oral or intravenous analgesics.

Bleeding was uncommon, occurring in 5.1% (2/39) of the head/neck group and 4.2% (1/24) of the body/tail group (*P =* 1.000), all managed with pharmacological hemostasis and, when indicated, blood transfusion (Grade 1-2). Ascites developed in 7.7% (3/39) and 4.2% (1/24) of patients, respectively (*P =* 1.000), and was successfully managed with albumin supplementation or percutaneous drainage (Grade 1-2).

Elevated serum bilirubin was observed in 12.8% (5/39) of the head/neck group versus 4.2% (1/24) of the body/tail group (*P =* 0.394). Although this difference did not reach statistical significance, the numerically higher rate in the head/neck group is anatomically plausible given the proximity of these tumors to the common bile duct. All cases were Grade 1–2 and resolved with conservative management, including supportive medications such as ursodeoxycholic acid and ademetionine, without requiring biliary drainage or stent revision. The median time for bilirubin normalization was 38 days (range, 25-63) in the head/neck group and 35 day in the single affected body/tail patient.

Portal vein thrombosis occurred in one patient (4.2%) in the body/tail group and in no patients in the head/neck group (*P =* 0.381). This complication was classified as CIRSE Grade 4 and was managed with systemic anticoagulation. No other major vascular complications, duodenal perforations, or pancreatic fistulae were observed in either group.

### 3.4 Survival outcomes

**Figure 1:**
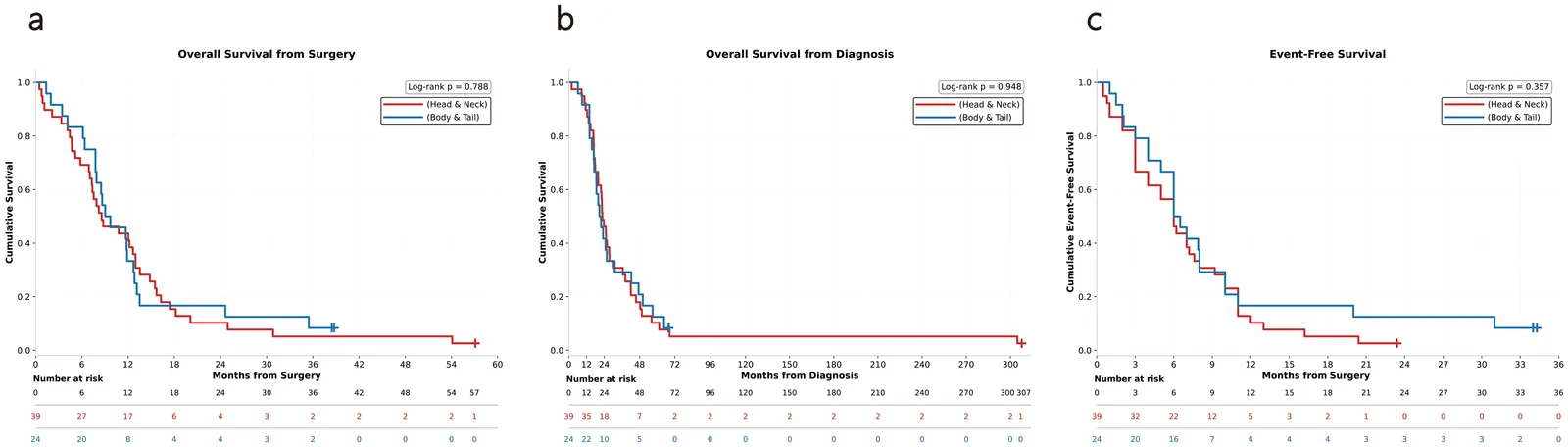
Kaplan-Meier survival curves. **(a)** Graphs show OS from the time of irreversible electroporation (IRE). **(b)** Graphs show overall survival (OS) from the time of diagnosis. **(c)** Graphs show Event-Free Survival (EFS) from the time of IRE. The head/neck group is displayed in red and the body/tail group in blue. Censored observations are marked with “+”. Log-rank P values are displayed in each panel.

Survival outcomes are summarized in **Table 4** and illustrated by the Kaplan-Meier curves in **Figure 1**. The median follow-up duration was 18.4 months (range, 1.5–54.1) for the head/neck group and 16.8 months (range, 2.4–35.5) for the body/tail group. The median OS from IRE surgery to death was 8.7 months (range, 0.47-54.10) in the head/neck group, compared to 9.37 months (range, 1.37-35.47) in the body/tail group. The log-rank test revealed no statistically significant difference in OS between the two groups (*P =* 0.788; **Figure 1a**). When OS was measured from the time of initial pancreatic cancer diagnosis to death, the median survival was 22.80 months (range, 1.90-304.70) for the head/neck group and 21.32 months (range, 6.17-64.67) for the body/tail group, again with no significant difference (*P =* 0.948; **Figure 1b**).

**Table 4:** Prognosis statistical analysis.

Characteristic	Head & neck	Body & tail	Log-rank p
Overall Survival from Surgery, months	8.7 (0.47,54.10)	9.37 (1.37,35.47)	0.788
Overall Survival from Diagnosis, months	22.80 (1.90,304.70)	21.32 (6.17,64.67)	0.948
Event-Free Survival, months	6.00 (0.50,34.70)	6.25 (1.00,31.00)	0.357

Except where indicated, data are expressed as median (minimum–maximum).

Event-free survival (EFS) from the time of IRE surgery, defined as the time to the first occurrence of local progression, distant metastasis, or death, showed a median of 6.00 months (range, 0.50-34.70) in the head/neck group and 6.25 months (range, 1.00-31.00) in the body/tail group (*P =* 0.357; **Figure 1c**). The overlapping EFS curves indicate that tumor location does not significantly influence the time to disease progression or recurrence following IRE. Kaplan-Meier curves for all three survival endpoints (OS from diagnosis, OS from IRE, and EFS from IRE) demonstrated substantial overlap between the head/neck and body/tail groups, with no significant separation at any time point. The number-at-risk tables beneath each curve confirmed adequate follow-up across time intervals.

Furthermore, an exploratory subgroup analysis confined to patients with non-metastatic advanced pancreatic cancer showed no statistically significant differences between the head/neck group and body/tail group in terms of overall survival from surgery (8.77 vs. 12.70 months, *P* = 0.321), overall survival from diagnosis (27.43 vs. 25.73 months, *P* = 0.466), and event-free survival (7.00 vs. 8.00 months, *P* = 0.242), aligning with the overall cohort findings.

To address potential confounding by systemic therapies, we performed stratified multivariable Cox proportional hazards regression for overall survival from diagnosis, adjusting for age, BMI, tumor size, distant metastasis, regional lymph node metastasis, and pre- and post-IRE treatments (chemotherapy, radiotherapy, surgical resection, and immunotherapy). The model was stratified by tumor location, BMI, and pre-IRE immunotherapy status to satisfy the proportional hazards assumption (global test *P* > 0.05). Post-IRE radiotherapy was marginally associated with improved survival (HR 0.380, 95% CI 0.127–1.136, *P* = 0.083), while distant metastasis showed a trend toward worse survival (HR 1.946, 95% CI 0.987–3.834, *P* = 0.055). Tumor location remained non-significant after full multivariable adjustment (stratified HR 1.212, 95% CI 0.687–2.137, *P* = 0.507). No other variable, including pre- and post-IRE chemotherapy or immunotherapy, was significantly associated with overall survival (all *P* > 0.05) (**Supplementary Table 1**).

### 3.5 Tumor marker dynamics

Changes in serum tumor markers at one-month post-IRE relative to preoperative levels were assessed in patients with available paired measurements. One month post-IRE, a decrease of >50% in CA19–9 was observed in 14 patients (35.9%) in the head/neck group and 7 patients (29.2%) in the body/tail group (*P =* 0.783). Similarly, a >50% reduction in CEA was achieved in 13 patients (33.3%) and 12 patients (50.0%) in the respective groups (*P =* 0.295). Neither tumor marker demonstrated a statistically significant differential response between the two anatomical groups, suggesting that the biological effect of IRE on tumor marker secretion does not vary by tumor location.

## 4 Discussion

This retrospective cohort study compared the safety, efficacy, and survival outcomes of percutaneous CT-guided IRE between patients with pancreatic head/neck tumors and those with body/tail tumors, representing, to the best of our knowledge, the first dedicated comparative analysis of IRE outcomes stratified by pancreatic location. Our principal finding is that IRE treatment outcomes (including perioperative complications, survival endpoints, and tumor marker dynamics) are comparable between the two anatomical subgroups, with no statistically significant differences observed in any of the primary or secondary outcome measures. These results provide important clinical evidence supporting the broad applicability of IRE across different anatomical subsites of the pancreas.

The finding that tumor location does not significantly affect IRE outcomes has important implications for clinical practice. In the surgical literature, pancreatic tumor location has long been recognized as a determinant of resectability, operative risk, and postoperative morbidity. Pancreaticoduodenectomy (Whipple procedure) for head tumors carries a distinct risk profile compared with distal pancreatectomy for body/tail tumors, including higher rates of pancreatic fistula, delayed gastric emptying, and biliary complications (18). However, our data suggest that for percutaneous IRE, these anatomical distinctions do not translate into differential procedural risk or efficacy. This may be explained by the non-thermal mechanism of action of IRE, which selectively ablates tumor cells while preserving the structural scaffolding of the extracellular matrix, including vascular and ductal structures (8, 9). The ability of IRE to spare the collagenous architecture of the bile duct, duodenal wall, and major vessels likely accounts for the comparable safety profile observed across pancreatic subsites (11).

The elevated serum amylase rate of approximately 60% observed in both groups merits discussion. The pancreas is an exquisitely sensitive organ, and even minimal perturbation of the pancreatic parenchyma — whether from needle puncture, electrical pulse delivery, or periablative edema — can trigger enzyme release. Our finding is consistent with the existing IRE literature; elevated amylase is the most commonly reported laboratory abnormality following pancreatic IRE, with rates ranging from 30-70% across published series (27). Critically, the vast majority of amylase elevations in our cohort and in the literature are self-limited and clinically inconsequential (CIRSE Grade 1-2), resolving within a median of 6 days with conservative management alone. The absence of any clinically significant pancreatitis or pancreatic fistula in either group reinforces the favorable pancreatic safety profile of IRE.

Our observed median OS from IRE surgery of 8.7 months in the head/neck group and 9.37 months in the body/tail group is comparable to the outcomes reported in the PANFIRE-2 multicenter Phase II trial, which documented a median OS of 17 months from diagnosis (corresponding to approximately 11 months from IRE) (14), and to the findings of Martin et al., who reported a median OS of 20 months from diagnosis in a prospective single-arm study (15). The OS from diagnosis in our cohort (22.80 months for head/neck vs. 21.32 months for body/tail) is somewhat longer than the 17-month median reported by the PANFIRE-2 trial, which may reflect differences in patient selection, the intensification of pre-IRE systemic chemotherapy regimens in the modern era (including the wider adoption of FOLFIRINOX and gemcitabine/nab-paclitaxel) (4, 6, 28).

The comparable EFS between the two groups (median 6.00 vs. 6.25 months, *P =* 0.357) is noteworthy. In the surgical literature, pancreatic body/tail tumors are associated with a higher propensity for peritoneal dissemination and a poorer prognosis compared with head tumors, partly attributable to later clinical presentation due to the paucity of early obstructive symptoms (29, 30). The absence of a significant difference in EFS in our IRE-treated cohort suggests that effective local tumor ablation may mitigate, at least in part, the biological disadvantage associated with body/tail tumor location by achieving adequate local disease control. Furthermore, the comparable systemic disease progression patterns between the two groups support the notion that IRE provides equivalent local control irrespective of the primary tumor site within the pancreas.

The tumor marker analysis revealed that neither CA19–9 nor CEA ratios at one month post-IRE differed between groups. CA19–9 is the most extensively studied biomarker in pancreatic cancer and has been shown to correlate with tumor burden, response to therapy, and prognosis (24, 25, 31). The observation that post-IRE CA19–9 dynamics do not vary by tumor location suggests that the magnitude of tumor ablation — and the consequent reduction in CA19-9-secreting tumor cell mass — is comparable across anatomical subsites. This finding aligns with the consistent procedural parameters (IRE delivery time, total procedure duration, and probe number) observed between the two groups, indicating that technical ablation adequacy is achievable irrespective of tumor location when IRE is performed by experienced operators under CT guidance. Specifically, IRE ablation of pancreatic head and neck lesions requires careful operation in narrow spaces beside the duodenum, bile ducts and vessels. By comparison, ablation for pancreatic body and tail involves longer retroperitoneal puncture paths prone to respiratory motion. Our results show that detailed CT-guided preoperative planning resolves region-specific anatomical obstacles and yields similar therapeutic efficacy across all lesions.

The only baseline characteristics that differed significantly between the two groups were body weight and BMI, both of which were higher in the body/tail group. This difference may reflect a potential selection bias towards lower BMI for pancreatic head cases in our clinical cohort. Importantly, this baseline variation in BMI did not translate into differential perioperative risk or adverse event rates between the two groups, indicating that IRE can be safely performed regardless of baseline BMI.

The emerging evidence supporting a synergistic interaction between IRE and immunotherapy warrants mention. Preclinical studies have demonstrated that IRE induces immunogenic cell death, characterized by the release of tumor-associated antigens, damage-associated molecular patterns (DAMPs), and pro-inflammatory cytokines that can prime adaptive anti-tumor immune responses (16). Xu et al. recently identified that insufficient IRE in residual tumors induces CCR2+ tumor-associated macrophage-mediated immunosuppression, which could be overcome by targeted CCR2 antagonism (32). In a mouse orthotopic pancreatic cancer model, IRE combined with intratumoral CD40 agonistic antibody stimulated systemic immune responses that inhibited liver metastasis, with 3 of 9 mice achieving complete bilateral tumor regression (33). Clinically, the CROSSFIRE trial demonstrated that IRE induced more pronounced systemic immunomodulation compared with stereotactic ablative body radiotherapy (SABR), including a significant reduction in activated regulatory T cells (aTregs, *P =* 0.033) and improved distant tumor-free survival (HR 0.38, 95% CI 0.17-0.85, *P =* 0.01) (34). A recent meta-analysis comparing IRE combined with immunotherapy versus IRE alone for locally advanced pancreatic cancer suggested a potential progression-free survival and OS benefit for the combination approach (35). In our cohort, most patients received post-IRE chemotherapy, with a subset also undergoing post-IRE immunotherapy or radiotherapy, reflecting the growing clinical adoption of multimodal treatment strategies.

Several limitations of the present study should be acknowledged. First, this is a retrospective, single-center study with inherent selection biases despite the use of a consecutive enrollment strategy.r Due to the relatively small sample size (39 vs. 24), the statistical power to detect subtle differences between groups may be limited. Thus, the absence of statistically significant differences (*P* > 0.05) should be interpreted as a lack of detectable variance in our cohort rather than definitive proof of absolute equivalence. Future multi-center studies with larger sample sizes may help identify independent prognostic factors that remained undetected in the present dataset. Second, the generalizability of our findings may be constrained by the single-center setting and the specific patient selection criteria applied at our institution. Additionally, the baseline BMI profile of our cohort is significantly lower than that typically seen in Western populations, as many Chinese patients present with severe cancer-induced cachexia, anorexia, and weight loss at initial diagnosis; this disparity should be recognized as a limitation when extrapolating our results to Western cohorts. Our cohort’s heterogeneity, including a mix of locally advanced and recurrent diseases, as well as the presence of distant metastases (43.6% head/neck; 50.0% body/tail), introduces potential confounding factors, as overall survival was heavily influenced by heterogeneous subsequent therapies rather than local IRE efficacy alone. To address this potential confounding, our exploratory subgroup analysis restricted to patients with non-metastatic locally advanced pancreatic cancer demonstrated consistent survival and progression profiles between the two groups, further reinforcing that anatomical location does not significantly impact IRE efficacy even in a highly homogeneous staging cohort. Consequently, this clinical confounding masks location-specific outcomes, requiring future prospective trials strictly limited to homogenous, non-metastatic advanced pancreatic cancer cohorts. Third, the heterogeneity in pre-IRE and post-IRE systemic therapies, including variations in chemotherapy regimens, immunotherapy agents, and the timing of these treatments, introduces potential confounding that cannot be fully adjusted for in a retrospective design. To address potential confounding by heterogeneous systemic treatments and clinical baselines, we performed a stratified multivariable Cox proportional hazards regression model with stratification by tumor location, BMI, and pre-IRE immunotherapy status (**Supplementary Table 1**). After adjusting for age, tumor size, metastatic status, and various pre- and post-IRE systemic therapies, these results indicated that the observed survival equivalence between anatomical subsites was not significantly influenced by the adjusted covariates. The stratified approach accounts for potential baseline differences between head/neck and body/tail tumors, ensuring that the comparison of other prognostic factors is performed within each location stratum. While restricted by our sample size, these adjusted findings suggest that the observed survival equivalence between anatomical subsites was unlikely to be solely driven by systemic treatment imbalances. Furthermore, EFS in our study included distant metastasis and all-cause death in addition to local progression. While local progression-free survival (LPFS) specifically reflects the direct efficacy of local IRE, EFS was adopted as a composite secondary endpoint to comprehensively capture overall systemic disease control and clinical benefit in this advanced cohort. Fourth, detailed dosimetry data, including the actual electric field distribution and the precise ablation zone volumes, were not systematically recorded, precluding a quantitative assessment of the relationship between ablation adequacy and tumor location. Lastly, quality-of-life assessments and patient-reported outcome measures were not collected, limiting our ability to comment on the functional impact of IRE according to tumor location.

Despite these limitations, this study addresses an important and clinically relevant question that has not been previously examined in a dedicated fashion. The consistent absence of statistically significant differences across all safety and efficacy endpoints provides a reasonable level of confidence that IRE outcomes are not disproportionately influenced by pancreatic tumor location. While systemic therapy remains the primary driver of survival in patients with advanced disease, IRE serves as a feasible and safe tool for achieving regional tumor control. Future prospective trials are warranted to confirm these findings and to establish standardized patient selection algorithms for IRE in the management of pancreatic cancer.

## 5 Conclusion

In this single-center retrospective cohort of 63 patients, no statistically significant differences were observed in procedural safety, complication rates, survival endpoints, or tumor marker dynamics between pancreatic head/neck and body/tail tumors following percutaneous CT-guided IRE. While limited by sample size, these preliminary findings suggest that tumor location alone does not inherently compromise procedural feasibility or short-to-medium-term outcomes. Consequently, anatomical location should not be viewed as an absolute contraindication for IRE; clinical decision-making should instead rely on multidisciplinary evaluation, patient performance status, and technical feasibility. Given the limited statistical power of this cohort, larger prospective, multicenter studies are essential to definitively validate the equivalence of treatment outcomes across anatomical subsites.

## Data Availability

The original contributions presented in the study are included in the article/**Supplementary Material**. Further inquiries can be directed to the corresponding authors.

## Supplementary material

Supplementary Figure 1 Flow diagram showing the selection process of patients with pancreatic tumors. The flowchart illustrates the exclusion of 43 cases from the initial cohort of 106 patients who underwent CT-guided irreversible electroporation (IRE), resulting in the final analyzed population of 63 eligible patients.
